# Binocular field configuration in owls: the role of foraging ecology

**DOI:** 10.1098/rspb.2023.0664

**Published:** 2023-10-18

**Authors:** Simon Potier, Alexandre Roulin, Graham R. Martin, Steven J. Portugal, Vincent Bonhomme, Thierry Bouchet, Romuald de Romans, Eva Meyrier, Almut Kelber

**Affiliations:** ^1^ Department of Biology, Lund University, Sölvegatan 35, Lund S-22362, Sweden; ^2^ Les Ailes de l'Urga, 72 rue de la vieille route, 27320 Marcilly la Campagne, France; ^3^ Department of Ecology and Evolution, University of Lausanne, Biophore 1015, Switzerland; ^4^ School of Biosciences, University of Birmingham, Edgbaston, Birmingham B15 2TT, UK; ^5^ Department of Biological Science, School of Life and Environmental Sciences, Royal Holloway University of London, Egham, Surrey TW20 0EX, UK; ^6^ ISEM, Univ Montpellier, CNRS, EPHE, IRD, 34095 Montpellier, France; ^7^ Équipe Dynamique de la biodiversité, anthropo-écologie, Place Eugène Bataillon - CC065, 34095 Montpellier Cedex 5, France; ^8^ Académie de Fauconnerie, SAS Puy du Fou France, 85500 Les Epesses, France; ^9^ Espace Rambouillet, Office National des Forêts, route du coin du bois, 78120 Sonchamp, France; ^10^ Les Aigles du Léman, Domaine de Guidou, 74140 Sciez sur Léman, France

**Keywords:** binocular vision, owls, raptors, foraging, morphometrics

## Abstract

The binocular field of vision differs widely in birds depending on ecological traits such as foraging. Owls (Strigiformes) have been considered to have a unique binocular field, but whether it is related to foraging has remained unknown. While taking into account allometry and phylogeny, we hypothesized that both daily activity cycle and diet determine the size and shape of the binocular field in owls. Here, we compared the binocular field configuration of 23 species of owls. While we found no effect of allometry and phylogeny, ecological traits strongly influence the binocular field shape and size. Binocular field shape of owls significantly differed from that of diurnal raptors. Among owls, binocular field shape was relatively conserved, but binocular field size differed among species depending on ecological traits, with larger binocular fields in species living in dense habitat and foraging on invertebrates. Our results suggest that (i) binocular field shape is associated with the time of foraging in the daily cycle (owls versus diurnal raptors) and (ii) that binocular field size differs between closely related owl species even though the general shape is conserved, possibly because the field of view is partially restricted by feathers, in a trade-off with auditory localization.

## 1. Background

Visual systems can determine the spatial position of a light source at any position within an animal's field of view and vision is, therefore, the crucial sense for many animals in the conduct of their key daily tasks such as foraging, mating and navigating [1]. In birds, binocular vision (the portion of the world around an animal's head viewed simultaneously by both eyes) may have important functions in operations such as controlling the direction of travel, positioning the bill tip or feet and timing when to make contact with a target [2]. Interestingly, closely related avian species have been shown to differ in the size of their binocular field, highlighting that ecological traits, rather than phylogeny, may be a major driver of binocular field configuration in birds [2,3].

Foraging appears to be a particularly important ecological trait in explaining variation in binocular overlap [3]. A wider binocular field is found in ibises (Threskiornithidae), whose foraging is primarily guided by visual cues compared to those whose foraging is guided by tactile cues [4], suggesting that binocular vision is essential for accurate positioning of the bill. Accipitriformes with different foraging tactics differ in the extension and shape of their binocular field, with species that forage on flying prey having a binocular field that is elongated and rectilinear, which may be advantageous to control foot positioning for catching prey that can escape in three dimensions [5]. Similarly, the relatively large binocular field of falcons (Falconidae) has been found to be of great importance while pursing and catching prey [6].

Despite growing evidence for the importance of binocular vision for foraging, owls (Strigiformes) remain comparatively understudied. Most owls are considered to be primarily nocturnal and many species are exclusively night-active (e.g. woodland owls), but some are crepuscular and even sometimes diurnally active (e.g. open habitat owls), and diet and foraging can be highly variable [7,8]. Thus, they present an excellent taxon in which to investigate binocular field variation. While the visual systems of owls are adapted in various ways to nocturnal activity [3,9], some species are able to forage by hearing alone [10–13]. This hearing ability is due to their elaborate outer ears that are hidden beneath the feathers of the owls' characteristic facial disc [13–17], preventing a more lateral placement of the eyes. Therefore, together with their characteristic foraging strategies (nocturnal activity and predatory habit), the elaborate outer ears may lead to a greater degree of binocularity than is found in species without such outer ear structures [2]. This hypothesis has been supported by comparative data with other nocturnal birds that lack outer ears, such as plovers (Charadriidae) and oilbirds (*Steatornis caripensis*), and which have extensive lateral visual fields and narrow binocular fields [2].

To date, visual field configuration has been studied in only one owl species, Tawny Owls (*Strix aluco*) [18]. Owl species experience different visual conditions: while owls have generally similar foraging strategies (most of the species use a perch and pounce strategy), they differ in their diet and preferences for foraging habitat [8,19]. Owl species living in woodlands tend to need moonlight intensities to see well enough at night to avoid small obstacles, while species living in open habitats can hunt visually even under starlight [7,20]. Consequently, owl species living in these different habitats should rely differently on their visual systems and in the way that they employ non-visual information to guide foraging when light levels are particularly low [7]. It is, therefore, of considerable interest to understand whether owls differ in their visual field characteristics and whether any differences are associated with ecological traits, especially diverse foraging habitats.

Here, we have focused on the interspecific variation in the binocular field configuration of 23 owl species (22 Strigidae and 1 Tytonidae) and present new data on the visual fields of 22 species. This sample size allowed us to test a number of hypotheses that address the role of ecological traits [21] and allometry (body mass and eye axial length [22,23]) in generating interspecific differences in owl binocular fields, while considering phylogenetic relatedness. We investigated the interspecific variation in binocular field configuration using both unidimensional (maximum horizontal binocular overlap and vertical extent of the binocular field) and multidimensional (shape of the binocular field using a geometric morphometric analysis) approaches [5]. Finally, because the diets of owls and diurnal raptors (Accipitriformes, Falconiformes and Cathartiformes) are broadly similar but these taxa differ in the general timing of their foraging and other visual capabilities (see [24] for a review), we compared the binocular field configuration between owls and diurnal raptors. We hypothesized that owls would have a larger binocular field than diurnal raptors because of their more frontally placed eyes [2]. Within owls, similarly to diurnal birds of prey [5], we expected that species chasing prey that move in three dimensions (a diet based mainly on insects) have a broader binocular field than owls chasing prey that move in only two dimensions (a diet based mainly on ground-dwelling mammals).

## 2. Methods

### (a) Species and study locations

Experiments were conducted at four French falconry parks: Les Ailes de l'Urga (July 2020), Espace Rambouillet (August 2020), Le Grand Parc du Puy du Fou (September 2020) and le Domaine des Fauves (April 2021); see table S1 in the electronic supplementary material for species list, sample size and location. The individual birds we worked with were measured close to their aviaries and were returned to them promptly after data collection was completed. The data collected at these sites were combined with the published dataset on Tawny Owls *Strix aluco* [18] for comparison of visual fields across 23 species of owls.

Because owls share similar diets but use different times of foraging in the daily cycle, we compared owls to diurnal raptors from Accipitriformes, Cathartiformes and Falconiformes. We used the 23 owl species studied in this paper to compare with the 19 diurnal raptor species published in previous papers [5,27–32] as well as one unpublished dataset from one Lanner falcon *Falco biarmicus* measured by S.P. in 2016 at Les Ailes de l'Urga.

### (b) Visual field measurements

We used the ophthalmoscopic reflex technique to measure visual field characteristics in alert birds. This non-invasive procedure has been described extensively in multiple previous investigations of visual fields in birds [5,28,29,31]. In summary, each bird was held firmly for 15–20 min in a plastic restraining tube of the appropriate size to avoid any body movement. The bird's legs were lightly taped together (Micropore Surgical tape 1530/1B) and cushioned by foam rubber held between them. The head was held at the centre of a visual field apparatus (using a device that permits the eyes to be examined from known positions around the head) by specially manufactured steel and aluminium bill holders. The head was maintained in the apparatus approximately at the position held when the bird is at rest naturally. A different bill holder was used for each species to account for differences in bill size and shape. The surfaces of the holders were coated in cured silicone sealant to provide a non-slip cushioned surface. We held the bill in place with Micropore tape, making sure not to cover the nostrils. We took calibrated photographs of the head of each bird while held in the apparatus to determine eye positions within the skull and the horizontal separation between the centres of the two eyes.

The perimeter's coordinate system followed conventional latitude and longitude measures, with the equator aligned vertically in the median sagittal plane of the head (i.e. a vertical plane that divides the head symmetrically into left and right hemispheres). We used this coordinate system in the presentation of the results. We examined the eyes using an ophthalmoscope mounted against the perimeter arm with an accuracy of ± 0.5°. We measured the boundaries of the retinal projection from the positions that the eyes spontaneously adopted when they were fully rotated ‘forwards’ (converged for estimation of binocular area boundaries) and ‘backwards’ (diverged for estimations of the blind sector's boundaries) for the blind area behind the head. We did not measure the projection of the pecten and the degree of eye movements to reduce the time for which the animals were restrained. Furthermore, the amplitude of eye movements is very small in owls (less than 1° [33]) and no perceptible eye movements were seen in the studied species.

We corrected our data for viewing from a hypothetical viewing point placed at infinity (this correction is based upon the distance used in the measurements taken with the visual field apparatus and the horizontal separation of the eyes [18]). After the corrections, we constructed a topographical map of the visual field and its different components. These features included: monocular fields, binocular field, cyclopean field (the total field around the head produced by the combination of the monocular fields of both eyes) and blind areas above and behind the head. The limits of the visual field were determined at 10° intervals of elevation in an arc from directly behind the head and then to above the head up to down to 60° below the horizontal in front of the head. However, depending on the bill shape of a given species, the bill holder intruded to different extents into our view of the eyes at a specific elevation. Therefore, we did not record data at elevations where the bill holder was blocking our view and instead estimated the binocular field width as the mean value of the binocular field widths immediately above and below these elevations [4].

### (c) Phylogeny

**Figure 1.**
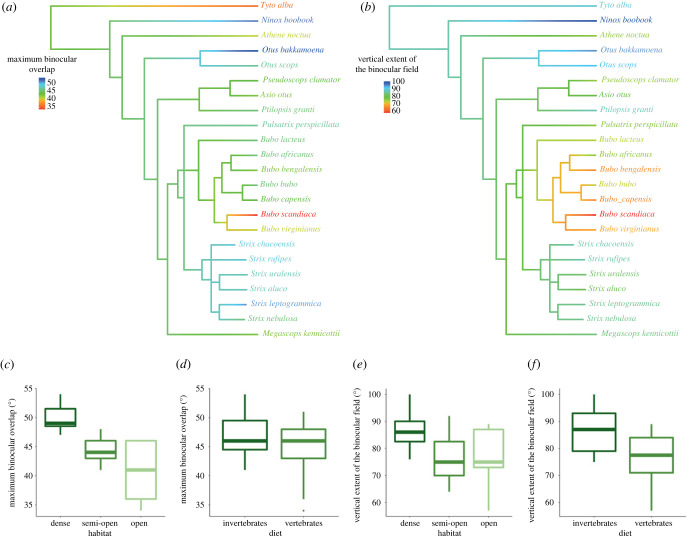
Phylogeny and ecological traits in owls. Phylogeny of owl species (*a,b*) included in this study. Branch colours indicate reconstructed ancestral states for visual field traits with (*a*) maximum binocular field width and (*b*) vertical binocular field extent. Colours at the tree tips represent the current states of these traits. (*c–f*) Visual field parameters with respect to ecological traits. (*c*) Maximum binocular overlaps as a function of habitat, (*d*) maximum binocular overlap as a function of diet (Western Screech Owl as invertebrate eater), (*e*) vertical extent of the binocular field as a function of habitat and (*f*) vertical extent of the binocular field as a function of diet (Western Screech Owl as invertebrate eater). (Boxplots: middle lines represent the median, boxes represent the IQR range from 25th (Q1) to 75th (Q3) percentiles, whiskers represent Q1 − (1.5 * IQR) and Q3 + (1.5 * IQR).

**Figure 2.**
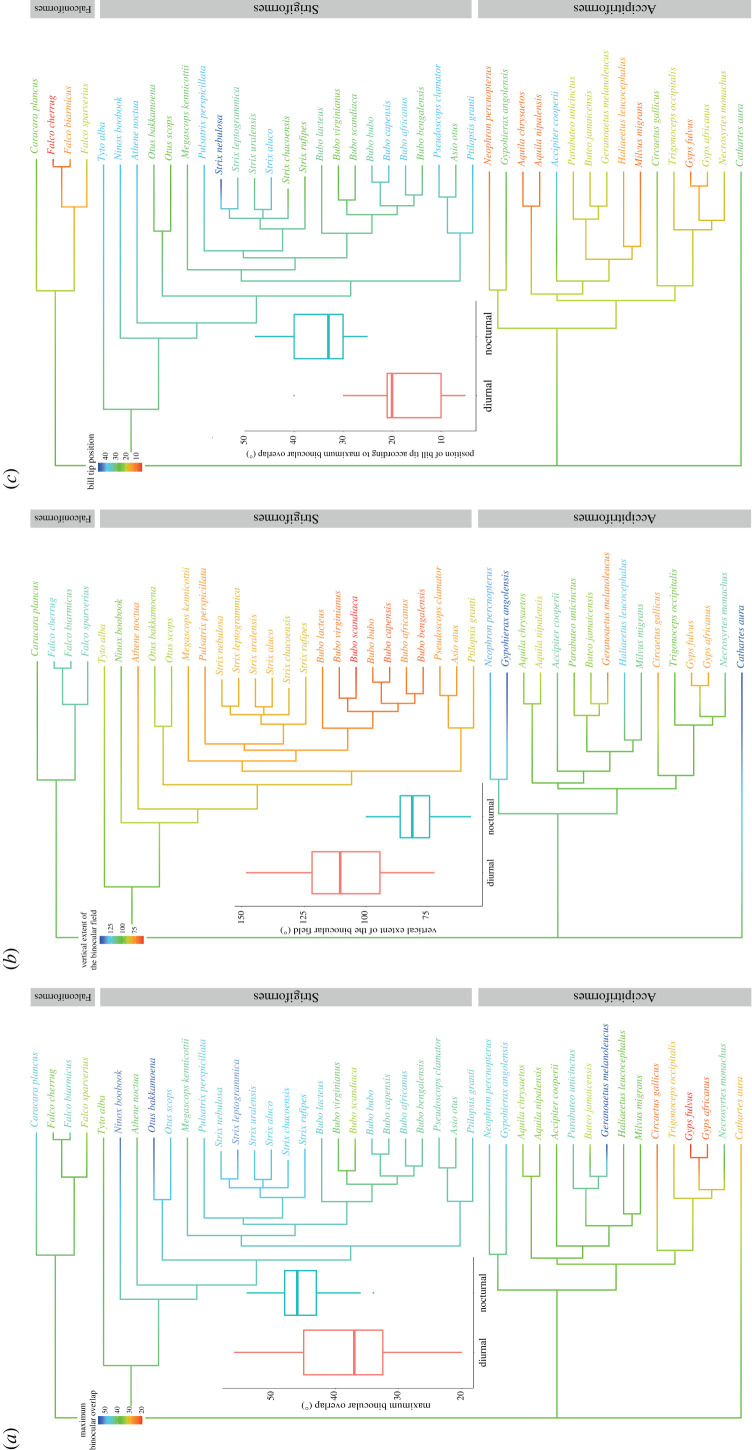
Binocular field and time of foraging in the daily cycle. Phylogeny and differences in binocular parameters between diurnal raptors and owls are presented. Branch colours indicate reconstructed ancestral states for visual field traits with (*a*) maximum binocular field width, (*b*) vertical binocular field extent and (*c*) position of bill tip according to maximum binocular overlap. Colours at the tree tips represent the current states of these traits. Boxplots: middle lines represent the median, boxes represent the IQR range from 25th (Q1) to 75th (Q3) percentile, whiskers represent Q1 − (1.5 * IQR) and Q3 + (1.5 * IQR).

We derived phylogeny from Jetz *et al*. [34]. This is the only available ‘complete’ species-level phylogenetic hypothesis for all birds (containing 9993 species), and based on this we downloaded 1000 ‘full’ trees containing the species dataset based on the ‘Hackett’ backbone [35] from www.birdtree.org. This procedure was used twice, once for owls only and once for both owls and diurnal raptors. We then generated maximum clade credibility trees for our analyses (figures 1 and 2).

### (d) Allometry

For owls only, we collected information about eye axial length from Ritland [26] and about body mass from Dunning [25].

### (e) Ecological traits

Data on diet composition (vertebrate eater or invertebrate eater) were extracted from Wilman *et al*. [19]. Species were classified as vertebrate or invertebrate eaters when more than 50% of diet composition correspond to one of the categorical groups (see electronic supplementary material for more information about diet composition). The Western Screech Owl *Megascops kennicottii* eats 50% vertebrates and 50% invertebrates. Therefore analyses were run twice considering this species as a vertebrate or invertebrate eater. Data on habitat density (dense, semi-open, open) were obtained from Tobias *et al*. [8]. Dense habitats represent lower or middle storey of forest, dense thickets, dense shrubland; open habitats comprise open shrubland, scattered bushes, parkland, low dry or deciduous forest, thorn forest; open habitats include desert, grassland, low shrubs, rocky habitats, cities (see [8] for more explanation).

Comparisons between diurnal raptors and owls were based only on their time of foraging in the daily cycle [8], not on other ecological traits.

### (f) Statistical analysis

We collected visual field data from three individuals per species (when possible, range: 1–3 individuals) and used averaged values for each species for the statistical analyses (see table S1). When comparing the shapes of visual fields in birds, we were limited to analysing the shape of the binocular field; we could not estimate all the limits of the lateral and blind portions due the aforementioned visual obstruction of the visual field apparatus [36].

Analyses were carried out using R v.4.0.4 (R Development Core Team 2021). To reconstruct the orthographic projection of the boundaries of the retinal fields of the two eyes for every species, we used the following packages: {ggplot2} [37], {ggforce} [38], {ggpubr} [39], {mapproj} [40] and {RVAideMemoire} [41]. To perform statistical analyses, we used the following packages: {Momocs} [42], {phytools} [43], {ggtree} [44], {phylolm} [45], {treeio} [46], {tidytree} [47], {TDbook} [48] and {GEIGER} [49].

We then compared the following parameters across species: (i) width of maximum binocular overlap, (ii) elevation at which maximum binocular overlap occurred according to bill tip position and (iii) vertical extent of the binocular field.

In addition, we compared the *shape* of the binocular field across species with a morphometric approach using outline analysis that aimed to translate *shapes* into quantitative variables to allow comparative analyses in a common multivariate framework [50]. The shape was defined as ‘the total of all information invariant under translations, rotations and isotropic rescaling’ [51]. From a visual field perspective, the morphometric analysis allowed the identification of variation in the shape of the binocular fields.

For the morphometric analysis, we calculated an elliptic Fourier transform (EFT) on the (*x*, *y*) coordinates of the binocular field outlines projected on a Cartesian plane. EFT turns *x*, *y* outline coordinates in two harmonic sums of trigonometric functions (one for the *x* coordinate and one for the *y* coordinate). Each harmonic is described by four harmonic coefficients (amplitude and phase for *x*, and the same for *y*). The EFT principle has been summarized elsewhere [50] and has been found to be the best approximation of an outline of a shape [52]. For Fourier-based approaches in morphometry, some rules are commonly used for the choice of the number of harmonics. Here, we followed: (i) the cumulated sum of squared harmonic coefficient as the harmonic power, (ii) the Euclidean distance between every two points of the reconstructed shape to the best possible reconstructed shape and (iii) visual inspection. Some minor editing (estimation of the lower bounds) was necessary to reconstruct the bottom section of the visual fields of three species because the apparatus did not allow observation of the eyes at the lowest elevations. In morphometric analyses, this minor editing has been shown to not affect the analysis [42].

We used PGLS models to phylogenetically correct slope and intercept estimates for relationship between variables. Pagel's lambda models were used to provide a quantitative estimate of the phylogenetic signal, based on deviations from a Brownian motion model. Model selection was performed using the ‘phylostep’ function from the {phylolm} package [45] following a Gaussian distribution, which select the model with the lowest Akaike information criterion (AIC). When habitat density had been selected, we performed a phylogenetic ANOVA (‘aov.phylo’ function from the {GEIGER} package [] based on Brownian Motion model) to estimate the effect of the whole independent factor.

Throughout the text, means are presented ± s.e.

## 3. Results

### (a) Visual fields parameters

**Figure 3.**
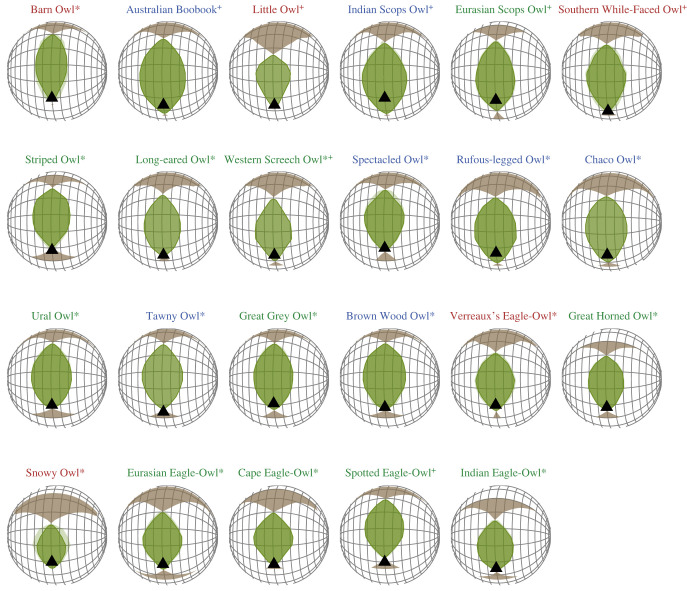
Orthographic projection of the boundaries of the retinal fields of the two eyes of owl species. A latitude and longitude coordinate system was used with the equator aligned vertically in the median sagittal plane. The bird's head is imagined to be at the centre of the globe (grid is at 20°intervals in latitude and 10° intervals in longitude). Green areas represent the binocular sector, white areas the monocular sectors and brown areas the blind sectors. The triangle indicates the direction of eye-bill tip projection. Light green sectors correspond to the standard deviation when different individuals were measured. Species names are coloured according to habitat (blue: dense; green: semi-open; red: open). Asterisk (*) indicates species that forage on vertebrates; plus (^+^) shows species that forage on invertebrates.

The visual fields of 22 species of owls measured in this study (listed in table S1 in the electronic supplementary material) are presented in figure 3. The width of the maximum binocular overlap across all 23 species—including the Tawny Owl measured in an earlier study—ranged from 34° in the Snowy Owl *Bubo scandiaca* to 54.5° in the Australian Boobook *Ninox boobook* (table S1 in the electronic supplementary material, figures 1–3). The vertical extent of the binocular field ranged from 57° in the snowy owl to 100° in the Australian Boobook *Ninox boobook* (table S1 in the electronic supplementary material, figures 1–3). Unfortunately, it was not possible to measure the limit of the visual field to the rear end of the head, at the eye horizon with precision. However, for all species, the width of the blind area behind the head in the horizontal plane was no larger than 180°. The more detailed study of the visual field in Tawny Owls reported the blind area to be 160° [18].

### (b) Unidimensional approach

Phylogeny explained the vertical extent of the binocular field (lambda = 0.97) but not the maximum binocular overlap (lambda = 1 × 10^−7^) or the elevation of maximum binocular overlap with reference to the direction of the projection of the eye-bill tip (lambda = 1 × 10^−8^).

We found a significant effect of diet (vertebrate versus invertebrate diets) for the vertical extent of the binocular field and the maximum binocular field overlap, but not the elevation of maximum binocular field overlaps according to bill tip position. This result was the same when the Western Screech Owl was considered as a vertebrate or invertebrate eater (table 1). Specifically, species foraging on invertebrates had larger binocular field overlap (Western Screech Owl as vertebrate eater: 47.7 ± 1.9 versus 44.6 ± 1.1° for invertebrate and vertebrate eaters, respectively; Western Screech Owl as invertebrate eater: 47.0 ± 1.7 versus 44.7 ± 1.1° for invertebrate and vertebrate eaters, respectively) and higher vertical extent of the binocular field (Western Screech Owl as vertebrate eater: 87.2 ± 4.2 versus 70.7 ± 2.2° for invertebrate and vertebrate eaters, respectively; Western Screech Owl as invertebrate eater: 86.6 ± 3.6 versus 76.3 ± 2.3° for invertebrate and vertebrate eaters, respectively) than those foraging on vertebrates (figure 1).

**Table 1.** Predictors (from selected models) of visual fields traits accounting for phylogeny in owls. Variable with *p*-values less than 0.05 are in italics.

Western Screech Owl *Megascops kennicottii* considered as a vertebrate eater						Western Screech Owl *Megascops kennicottii* considered as an invertebrate eater					
variable	term	estimate	s.e.	*t*	*p*	variable	term	estimate	s.e.	*t*	*p*
PC1	(intercept)	0.0732	0.0384	1.906	0.070	PC1	(intercept)	0.091	0.040	2.256	*0.035*
							diet [vertebrate]	−0.033	0.023	−1.427	0.168
PC2	(intercept)	−4.27e^−04^	0.008	−0.052	0.959	PC2	(intercept)	0.022	0.014	1.567	0.132
							diet [vertebrate]	−0.032	0.017	−1.908	0.070
maximum binocular overlap	(intercept)	52.595	1.427	36.865	*<0.001*	maximum binocular overlap	(intercept)	52.048	1.463	35.585	*<0.001*
	diet [vertebrate]	−3.621	1.351	−2.68	*0.015*		diet [vertebrate]	−2.867	1.34	−2.139	*0.046*
	habitat [semi-open]	−4.966	1.339	−3.708	*0.001*		habitat [semi-open]	−5.295	1.405	−3.768	*0.001*
	habitat [open]	−9.823	1.633	−6.014	*<0.001*		habitat [open]	−9.728	1.72	−5.655	*<0.001*
vertical extent	(intercept)	97.069	6.519	14.891	*<0.001*	vertical extent	(intercept)	96.596	6.355	15.199	*<0.001*
	diet [vertebrate]	−9.715	3.431	−2.832	*0.011*		diet [vertebrate]	−9.415	3.26	−2.888	*0.009*
	habitat [semi-open]	−3.004	2.811	−1.069	0.299		habitat [semi-open]	−3.687	2.806	−1.314	0.204
	habitat [open]	−9.066	3.991	−2.271	*0.035*		habitat [open]	−9.046	3.964	−2.282	*0.034*
elevation of maximum binocular field overlap	(intercept)	34.288	1.305	26.271	*<0.001*	elevation of maximum binocular field overlap	(intercept)	34.288	1.305	26.271	*<0.001*

Habitat has been selected for both the vertical extent of the binocular field and the maximum binocular field overlap, but not for the elevation of the maximum binocular field overlap with respect to eye-bill tip projection (table 1). However, testing the significance of the whole independent factor showed a significant effect of habitat on maximum binocular field overlap (d.f. = 2, *F* = 8.18, *p* = 0.01) but not for the vertical extent of the binocular field (d.f. = 2, *F* = 1.50, *p* = 0.31). Therefore, the pairwise differences between habitat cannot be regarded as significant for vertical extent of the binocular field. Binocular field overlap was smallest in species living in open habitats and largest in species living in dense habitats (open = 40.6 ± 2.5°, semi-open = 44.6 ± 0.6°, dense = 50.0 ± 0.9°; electronic supplementary material, tables S2 and S3; figure 1).

Neither body mass nor axial length of the eye was retained in our model selection, nor did they explain variation in the vertical extent of the binocular field, maximum binocular field overlap or elevation of maximum binocular field overlap according to the position of the eye-bill tip projection.

### (c) Multidimensional approach

**Figure 4.**
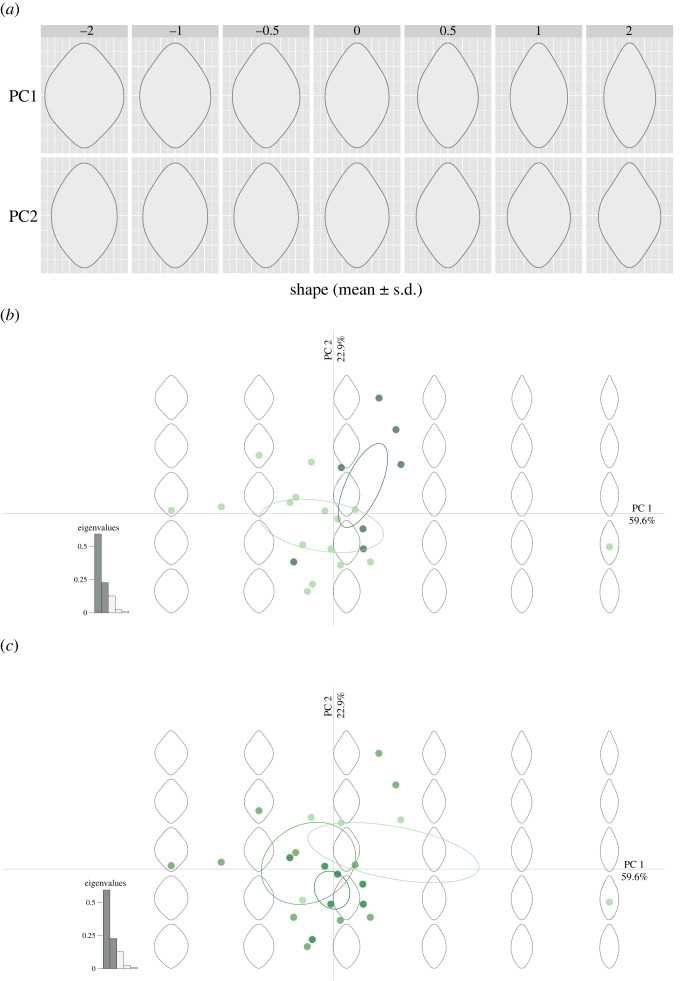
Principal component analysis of owl visual field data. (*a*) Aspects of binocular field shapes captured by the first two principal components. Binocular field shape with respect to (*b*) diet (dark green: invertebrate eater, light green: vertebrate eater; Western Screech Owl as invertebrate eater) and (*c*) habitat (dark green: dense, medium green: semi-open, light green: open).

Variation in binocular field shape is explained by the first two principal components (PCs), which gathered 82.5% of the total variance (59.6% for PC1, eigenvalue: 0.595; 22.9% for PC2, eigenvalue: 0.229) (figure 4). Positive PC1 scores represent a narrower binocular field shape in the horizontal extension (figure 4*a*). Positive PC2 scores represent a protrusion shape (at the lower and upper edge) of the binocular field (figure 4*a*). Variation in PC1 and PC2 scores did not reflect the phylogenetic signal (lambda < 1 × 10^−4^). Species with different diets (both when the Western Screech Owl was considered as vertebrate and as invertebrate eater), habitat or body mass did not vary significantly in PC1 or PC2 scores (table 1), which reflects the generally similar binocular field shape among owls (figure 4*b,c*).

### (d) Comparison between diurnal raptors and owls

Phylogeny explained the vertical extent of the binocular field (lambda = 0.989) and partially explained the maximum binocular overlap (lambda = 0.604), but did not explain the elevation of maximum binocular overlaps according to bill tip position (lambda = 1.740 × 10^−8^).

Diurnal raptors and owls did not differ significantly in the maximal binocular field width (diurnal raptors: 37.4 ± 2.0°, owls: 45.4 ± 1.0°; estimate = 6.475, s.e. = 6.054, *t* = 1.070, *p* = 0.291) nor in the vertical extent of their binocular field after controlling for phylogeny (diurnal raptors: 108.3 ± 5.1°, owls: 79.4 ± 2.2°; estimate = −35.164, s.e. = 20.295, *t* = −1.733, *p* = 0.091) (figure 2*a,b*). However, diurnal raptors and owls differed in the position of their bill tips (estimate = 15.623, s.e. = 2.266, *t* = 6.896, *p* < 0.001). Diurnal raptors had their bill tip closer to the position of their maximum binocular field width than owls (difference between the positions of maximum binocular field and bill tip: 18.7 ± 2.0°versus 34.3 ± 1.3° for diurnal raptors and owls, respectively; figure 2*c*).

**Figure 5.**
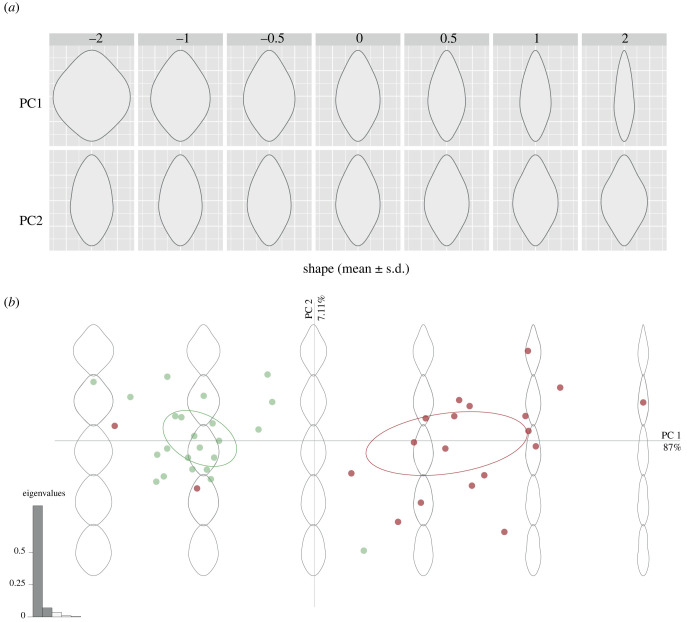
Principal component analysis of owls and diurnal raptors visual field data. (*a*) Aspects of binocular field shapes captured by the first two PCs. (*b*) Binocular field shape with respect to time of foraging in the daily cycle (diurnal raptors in red, owls in green).

Variation in binocular field shape is explained by the first two PCs, which gathered 94.11% of the total variance (87.1% for PC1, eigenvalue: 0.871; 7.01% for PC2, eigenvalue: 0.070) (figure 5). Positive PC1 scores represent a narrower binocular field shape in the horizontal extension (figure 5*a*). Positive PC2 scores represent a protrusion shape (at the lower and upper edge) of the binocular field (figure 5*a*). We did not find a phylogenetic signal for the variation in PC1 (lambda < 10^−7^) and PC2 (lambda < 10^−8^). Beyond this, diurnal raptors and owls differ in their PC1 scores, with diurnal raptors having a more elongated binocular field shape than owls (estimate = −0.265, SE = 0.030, *t* = −8.889, *p* < 0.001; figure 5*b*). No difference was found between diurnal raptors and owls for PC2 scores (estimate = 5.138 × 10^−3^, s.e. = 0.014, *t* = 0.353, *p* = 0.726; figure 5*b*).

## 4. Discussion

Overall, we found that the studied owls differ in the size of their binocular region according to their diet and the habitat in which they forage. Species foraging on invertebrates and species foraging in dense habitats have a larger (wider and longer) binocular field than those foraging on vertebrates and those foraging in open habitats. Besides this size variation all studied owl species share a similar binocular field shape, which is less elongated than that of diurnal species. Our study is based on very few individuals per species, but visual field measurements are relatively repeatable among species [53]. While this is likely a general pattern, more than 23 species of owls and 20 species of diurnal raptors may need to be studied to be entirely sure.

### (a) Size, but not shape, of the binocular field of owls is influenced by ecological traits

Binocular field configuration in owls appears to be unique and different from findings in other birds tested so far (large broad frontal binocular field and bill tip projection just outside or at the extreme inferior limit of the binocular field [54]). However, we still found variation in the binocular field of owls. While all owl species shared a similar binocular field shape, binocular field size (vertical extent and maximum binocular field width) differed with diet and habitat, highlighting that variation was largely dictated by ecological traits rather than allometry and phylogeny. A possible exception is the Barn Owl *Tyto alba*, which was the only species belonging to Tytonidae included in the study. Barn Owls had a different binocular field from the other owl species, reflecting the need to study more species belonging to this family to determine whether this trait is consistent in the Tytonindae.

Visual field variation dictated by foraging has been found within other groups of closely related bird species, with species needing accurate bill and/or foot positioning having larger binocular fields (reviewed in [3]). We found here that owls foraging on invertebrates had larger binocular fields than those foraging on vertebrates (mostly mammals). A similar pattern has been found in diurnal raptors [5]: species foraging on prey moving in three dimensions (foraging for aerial and aquatic prey) have a more rectilinear binocular field shape than species foraging on prey moving primarily in two dimensions only (foraging on terrestrial prey). However, most owl species foraging on invertebrates do not forage on the wing (perhaps with the exception of the Long-eared Owls *Asio otus*) [8]. Thus, the foraging tactics should not differ much between species foraging on vertebrates and invertebrates. A possible reason for the larger binocular fields in insectivorous owl species might be that these species may mostly use visual information when hunting, while owl species foraging on vertebrates (mammals) may be more dependent on sound cues. A recent study showed that species foraging on mammals may depend more on hearing during foraging because their relatively greater facial disc facilitates sound localization, and longer combs on the leading edges of primary feathers produce silent flight [55]. For instance, Great Grey Owls *Strix nebulosa*, like other owls foraging on mammals, may plunge through snow to capture their prey without any visual cues [56]. Therefore, the species foraging on invertebrates may be more likely to localize prey by vision, which requires a larger binocular field (but see [57]). This may highlight again the trade-off between hearing and vision in owls.

Species foraging in dense habitats had wider binocular fields than species foraging in more open habitats. Large binocular fields may be essential to avoid obstacles, as has been shown in humans [58,59]. Therefore, owls living in dense habitats may need a larger binocular field to enable them to move over and around obstacles. It has also been proposed that large binocular fields become more important as light levels decrease; extracting information from optic flow-fields in the binocular region by both eyes may improve the signal-to-noise ratio and thus increase the reliability of vision in dim light conditions [18,60]. Because species living in dense habitats experience lower light intensities [61], these species might also need larger binocular fields. Large binocular fields are in part associated with larger eyes in owls [62,63], suggesting that species living in dense habitats may have larger eyes relative to body mass. This is in part confirmed by this study, with the ratio of eye axial length and body mass differing among species from different habitats (0.075 ± 0.021, 0.055 ± 0.022 and 0.055 ± 0.029 for dense (*n* = 5), semi-open (*n* = 6) and open (*n* = 3) habitats, respectively). However, as the sample size was too low for each habitat type, no statistical tests could be performed and further studies are needed.

### (b) Binocular field shape differs between owls and diurnal raptors

In this study, we found that the shape, but not the size, of the binocular visual field significantly differs between owls and diurnal raptors; diurnal raptors had more elongated binocular fields than owls. As suggested previously [2,18,54], owls may have a unique configuration of the binocular region among birds (visual field type 3 [54]).

It has been shown that owls have higher acuity in the binocular than in the monocular visual field [64], unlike other bird species (but see [65]). In birds, the presence or absence of a fovea (an invagination in the retina with high photoreceptor density) is related to ecological traits [66]. Many birds possess one fovea, others have two (e.g. diurnal raptors except scavengers [67,68]) and some lack a fovea entirely. Most owls possess one fovea, but unlike uni-foveate diurnal birds whose fovea is placed centrally in the retina, their fovea is placed temporally [69–71] and, thus, looks forwards into the binocular field. In Barn Owls (Tytonidae), which do not possess a fovea [70], the binocular field region is considerably narrower than in owls from the family Strigidae. This suggests a link between the presence of a temporal fovea and binocular field width in owls. However, in other species, the temporal fovea may not be involved in binocular vision [72], highlighting the importance of understanding the role of the temporal fovea for frontal vision.

Compared to other birds, owls have numerous binocularly driven neurons [73], which may be a result of their combined use of vision and hearing, using their uniquely elaborate outer ears for accurate auditory localization of prey [11,14,62]. The more frontal position of owl eyes (even though they are still laterally placed [2]) likely results from selection for large eyes with high visual sensitivity [62,63] combined with selection for large outer ear structures [14]. Such elaborate outer ear structures are not found in other nocturnal birds (such as plovers and oilbirds), whose eyes are placed more laterally and therefore have narrower binocular fields [2]. It has been shown that the facial disc in owls serves as a sound amplifier for auditory cues that are used while foraging [12]. However, as shown previously in the only owl species tested before (Tawny Owls [18]), the margin of the binocular field coincides with the feathering of the facial disc for all species tested in this study (S Poitier 2020–2021, personal observations). This may highlight a possible trade-off between two sensory modalities in owls: vision and hearing.

We found a significant difference between diurnal raptors and owls in the projection of the bill tip direction in the binocular field. In owls, the bill tip fell outside of or at the extreme inferior limit of the binocular field, while in diurnal raptors, the bill tip projects close to the region of maximum binocular field width. This suggests that the binocular field might not serve bill tip positioning in owls, but rather guides the positioning of the feet when the bird is pouncing on prey (which is also essential for diurnal raptors [28]). The accommodative power of the cornea is much lower in owls than in diurnal raptors ([74–76], reviewed in [24], but see the exception of American Barn Owls *Tyto furcata* [76]), suggesting that they may not be able to focus on objects that are very close to the eyes. Instead, similar to other nocturnal birds, owls have elaborate bristles around the beak that are sensitive to vibrotactile signals [77–79]. These facial bristles have been suggested to play a key role in prey handling [78]. Owls also often handle prey with their feet to bring it towards the bill tip. Therefore, judging accurate bill tip position by vision might not be as important for owls because they may use other sensory abilities or behaviours to position their prey after capture.

In summary, the binocular field configuration of owls may have evolved together with hearing and mechanoreception, demonstrating multimodally guided foraging in owls.

## 5. Conclusion

Owls have a significantly different binocular field shape from diurnal raptors. Bill position within the binocular field also differs significantly between these two taxa. We suggest that these differences are related to the use of both vision and hearing to guide foraging in owls, while diurnal raptors are primarily visually guided. Variation in binocular field size within owls was found to be correlated with differences in two ecological traits: habitat density and diet. Overall, our study highlights the importance of studying both the shape and the size of binocular fields. It also suggests that binocular field shape and size may reflect important adaptations to the sensory challenges of nocturnal and diurnal activity in birds of prey, as well as inter-species differences in foraging behaviour and ecology, as reported previously in diurnal raptors [5].

## Data Availability

The data are provided in electronic supplementary material [80].
